# Synthesis, microstructure, and magnetic properties of monosized Mn_*x*_Zn_*y*_Fe_3 − *x* − *y*_O_4_ ferrite nanocrystals

**DOI:** 10.1186/1556-276X-8-530

**Published:** 2013-12-17

**Authors:** Hayoung Yoon, Ji Sung Lee, Ji Hyun Min, JunHua Wu, Young Keun Kim

**Affiliations:** 1 Department of Materials Science and Engineering, Korea University, Seoul 136-713, Republic of Korea; 2 Pioneer Research Center for Biomedical Nanocrystals, Korea University, Seoul 136-713, Republic of Korea

**Keywords:** Ferrite, Nanocrystal, Nanoemulsion, Ferrimagnetism, Superparamagnetism, 61.46. + w, 75.20.-g, 75.50.Gg

## Abstract

We report the synthesis and characterization of ferrite nanocrystals which exhibit high crystallinity and narrow size distributions. The three types of samples including Zn ferrite, Mn ferrite, and Mn-Zn ferrite were prepared via a non-aqueous nanoemulsion method. The structural, chemical, and magnetic properties of the nanocrystals are analyzed by transmission electron microscopy, X-ray diffraction, X-ray fluorescence, and physical property measurement system. The characterization indicates that the three types of ferrite nanocrystals were successfully produced, which show well-behaved magnetic properties, ferrimagnetism at 5 K and superparamagnetism at 300 K, respectively. In addition, the magnetization value of the ferrites increases with the increasing concentration of Mn.

## Background

Ferrite nanocrystals have been interestingly studied due to their tunable and remarkable magnetic properties such as superparamagnetism [1-3], as well as catalytic properties not existing in the corresponding bulk materials [4,5]. There have been extensive investigations on ferrite nanocrystals for potential applications in magnetic storage, ferrofluid technology, and biomedical fields from drug delivery, hyperthermia treatments, to magnetic resonance imaging [6-10].

A ferrite has the spinel structure basically constructed from face-centered cubic lattices formed by oxygen ions and assumes a general formula described as (M^2+^_1 − δ_Fe^3+^_δ_)_tet_[M^2+^_δ_Fe^3+^_2 − δ_]_oct_O_4_[11]. The element M in the formula can be a transition metal, like Mn, Co, and Zn. Moreover, the round and square brackets indicate the tetrahedral site (A site) and octahedral site (B site) created by oxygen ions, respectively. The subscription, δ, in the range from 0 to 1, represents the inversion parameter of the spinel structure. The parameter could be adjusted in terms of various factors, for example, synthesis methods, particle size, and heat treatments [12-18]. The ferrimagnetism of the ferrite is originated from the exchange energy between the A and B sites (A-B interaction) which is larger than other interactions (A-A, B-B). Since the A-B interaction has a negative value, the ions located in both sites have antiparallel orientations; consequently the net moments between both sites result in ferrimagnetism [19-23]. Therefore, possible variation of ion arrangements in the lattices may affect the magnetic properties of the ferrite.

In this study, we report the synthesis and characterization of Mn_*x*_Zn_*y*_Fe_3 − *x* − *y*_O_4_ ferrite nanocrystals, i.e., *x* = 0, *y* = 0.9 for Zn ferrite, *x* = 0.6, *y* = 0 for Mn ferrite, and *x* = 0.315, *y* = 0.45 for Mn-Zn ferrite via a nanoemulsion method. The structure, chemical, and magnetic properties of the nanocrystals were comparatively analyzed by transmission electron microscopy (TEM), X-ray diffraction (XRD), X-ray fluorescence (XRF) spectroscopy, and physical property measurement system (PPMS).

## Methods

The ferrite nanocrystals of the three types were synthesized by the nanoemulsion method with a biocompatible polymer [24,25]. The synthesis was performed by thermal decomposition of precursors including iron(III) acetylacetonate, manganese(II) acetylacetonate, and zinc(II) acetylacetonate hydrate. In the case of the Zn ferrite, the iron and zinc precursors were added at a molar ratio of 2:1. In the same manner, the iron and manganese precursors were added at a ratio of 2:1 for the Mn ferrite, while for the Mn-Zn ferrite, the iron, manganese, and zinc precursors were added at a ratio of 4:1:1. 1,2-Hexadecanediol and octyl ether were used as the reductant and the solvent, respectively. The completion of the reactions was achieved in the nanoreactors formed by poly(ethylene glycol)-*block*-poly(propylene glycol)-*block*-poly(ethylene glycol) (PEO-PPO-PEO) polymer surfactant. All chemicals were purchased from Sigma-Aldrich Corporation (St. Louis, Missouri, USA), except for octyl ether (Tokyo Chemical Industry Co., Ltd., Tokyo, Japan). The mixture was first heated to 120°C for 1 ~ 2 h, and then the temperature was raised rapidly to 280°C for refluxing. After 1 h of refluxing, the solution was air-cooled and washed with ethanol several times. The washed solution was subsequently centrifuged to precipitate the nanocrystals.

The crystal structures, particle sizes, and shapes of the nanocrystals were investigated by XRD (D/MAX-2500 V/PC; Rigaku Corporation, Tokyo, Japan) and TEM (JEM-2100 F; JEOL Ltd., Tokyo, Japan) including high-resolution transmission electron microscopy (HRTEM), while the chemical compositions of the nanocrystals were determined by an energy-dispersive spectroscopy (EDS) system in TEM and XRF (S2 PICOFOX; Bruker Corporation, Billerica, MA, USA). In addition, the magnetic behaviors of the nanocrystals were analyzed by a PPMS (Quantum Design Inc., San Diego, CA, USA).

## Results and discussion

The reactions were completed through the thermal decomposition of the appropriate precursors in the nanoreactors formed by the polymer molecules, resulting in high-quality nanoparticles as desired [24]. The use of the polymer, PEO-PPO-PEO, is distinctive, which has many merits and broad applications. In particular, the polymer is bio-friendly [25] and has an amphiphilic property [24], so the synthesized nanoparticles can be well dispersed in an aqueous solution without any additional surface modifications, which is especially benign for biomedical purposes [24].

**Figure 1:**
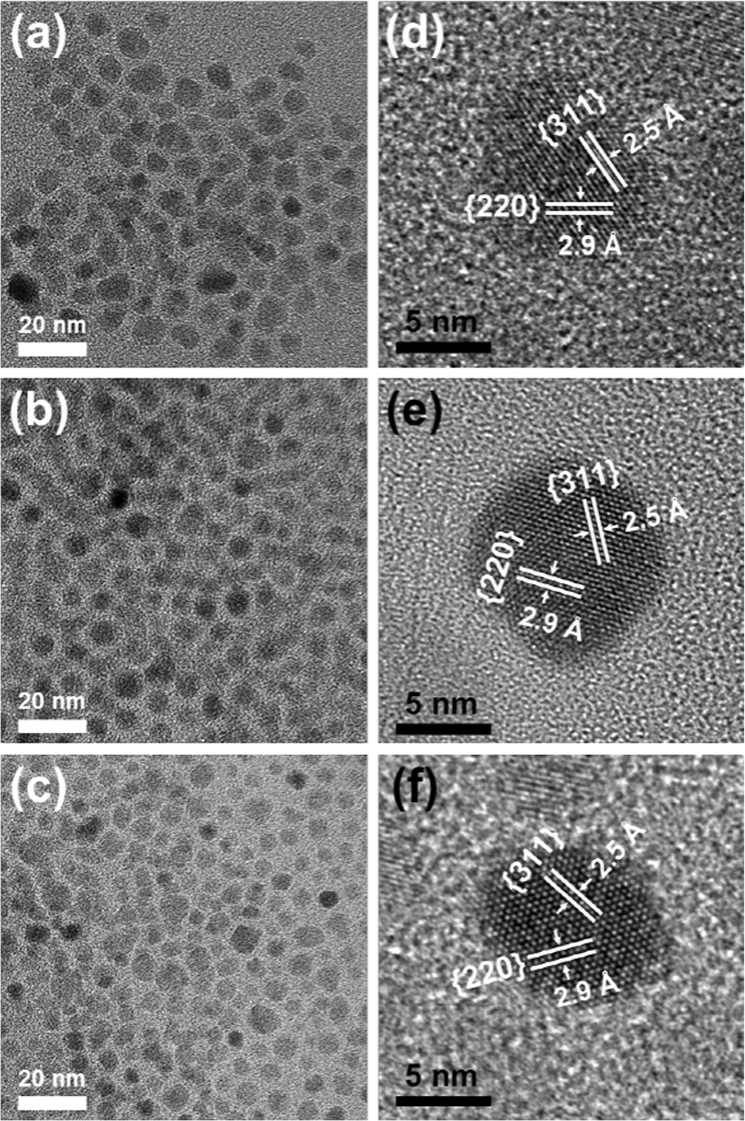
**TEM analysis of the ferrite nanocrystals.** TEM images of **(a)** Zn ferrite, **(b)** Mn ferrite, and **(c)** Mn-Zn ferrite. HRTEM images of **(d)** Zn ferrite, **(e)** Mn ferrite, and **(f)** Mn-Zn ferrite.

The TEM images in Figure 1a,b,c show the morphologies and particle sizes of the ferrite nanocrystals. In the images, the nanocrystals appear almost spherically shaped and monosized. The size distributions of the nanocrystals were obtained by size counting from the relevant TEM images, which were fitted well by Gaussian distributions, giving an averaged diameter and standard deviation of 7.4 ± 0.7 nm for Zn ferrite, 7.1 ± 0.9 nm for Mn ferrite, and 6.2 ± 0.8 nm for Mn-Zn ferrite, respectively. Figure 1d,e,f displays the HRTEM images for the corresponding ferrite nanocrystals showing highly crystalline characteristics. The individual lattices in the images are separately indexed to the projected (220) and (311) planes of the cubic spinel structure of ferrites.

**Figure 2:**
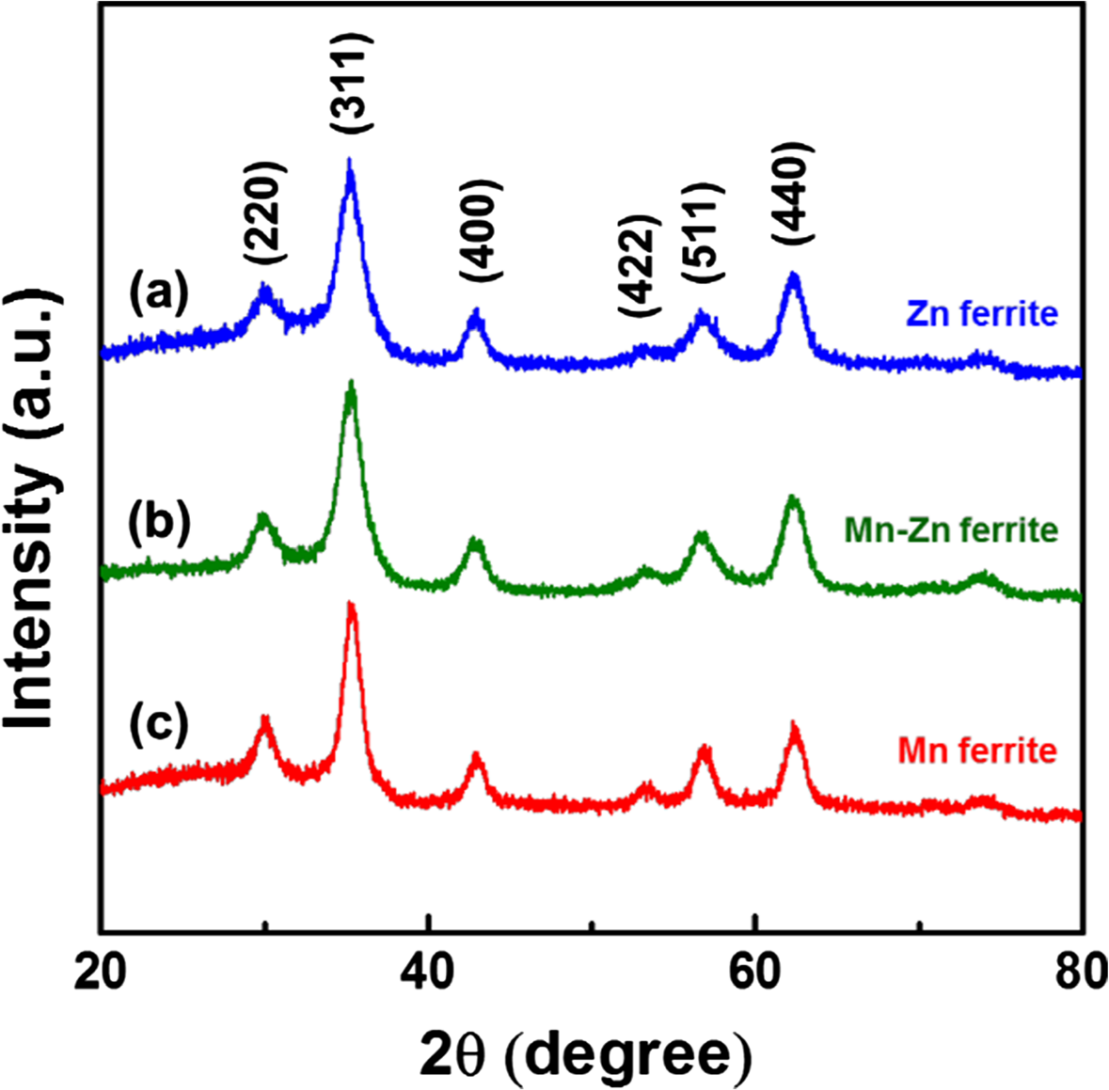
**XRD diffraction patterns for the ferrite nanocrystals. (a)** Zn ferrite, **(b)** Mn-Zn ferrite, and **(c)** Mn ferrite.

The structural information on the nanocrystals is further acquired by XRD analysis. Figure 2 illustrates the XRD patterns of the three types of the ferrite nanocrystals. All XRD diffractions show the typical peaks of the spinel structure, such as (220), (311), and (400), without any other unexpected peaks from by-products like MnO, ZnO, or other metal oxide forms. The results clearly indicate that all nanocrystals were properly synthesized in ferrite forms. Moreover, it is observable that the peaks in the XRD patterns are shifted to lower angles slightly as the concentration of Zn increases. For example, the positions of the (311) peaks are 35.41° for Mn ferrite, 35.28° for Mn-Zn ferrite, and 35.23° for Zn ferrite, separately. According to the Bragg's law, the reduced angle of the diffraction peaks originated from the increased lattice spacing. In fact, a Zn^2+^ ion has the radius of 0.88 Å, which is larger than the radius of an Fe^2+^ ion (0.75 Å) and Mn^2+^ ion (0.81 Å), so the increasing of Zn^2+^ ion substitution leads to the expansion of the lattice spacing. Consequently, the phenomenon as observed above corroborates that the Zn^2+^ and Mn^2+^ ions were successfully doped in the relevant ferrite nanocrystals.

Table 1 summarizes the chemical compositions of the ferrite nanocrystals analyzed by XRF and TEM-EDS. The XRF data report the atomic ratio of the nanocrystals in a large quantity, while the EDS data present the composition of a singular particle. Nonetheless, both data show a close match in the chemical composition. Compared with the precursor ratios, the XRF and EDS data reveal no substantial difference of Zn and Mn of the resultant nanocrystals from the one designed originally. Thus, the composition formulas are described as Zn_0.9_Fe_2.1_O_4_ for Zn ferrite, Mn_0.6_Fe_2.4_O_4_ for Mn ferrite, and Mn_0.3_Zn_0.5_Fe_2.2_O_4_ for Mn-Zn ferrite.

**Table 1:** Chemical compositions of the ferrite nanocrystals

		**Precursor molar ratio**	**XRF (at.%)**	**EDS (at.%)**
Zn ferrite	Fe	2	71.3	70.9
	Zn	1	28.7	29.1
Mn ferrite	Fe	2	77.7	79.7
	Mn	1	22.3	20.3
Mn-Zn ferrite	Fe	4	74.4	78.6
	Zn	1	15.2	11.8
	Mn	1	10.4	9.6

**Figure 3:**
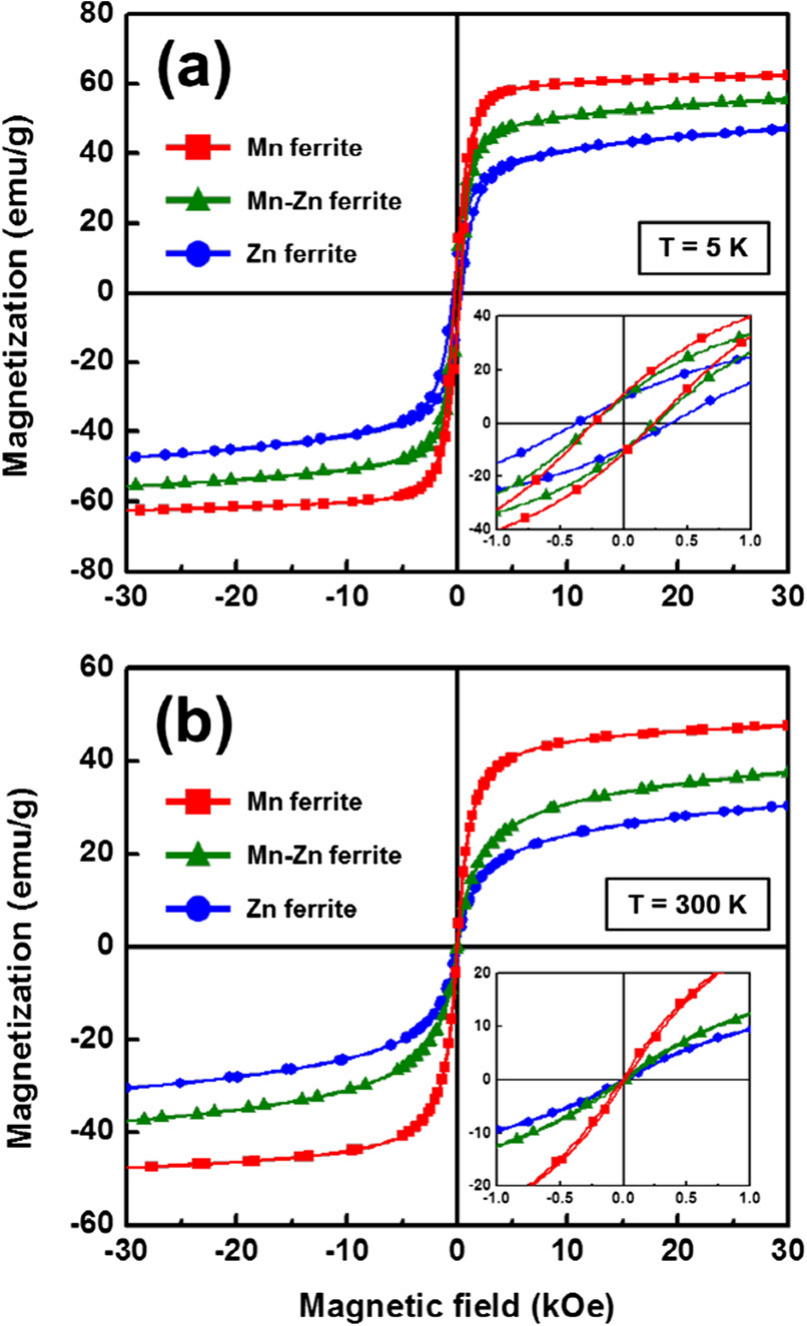
**Magnetic analysis of the ferrite nanocrystals. (a)** M-H hysteresis curves at 5 K and **(b)** 300 K.

Figure 3a,b records the hysteresis curves obtained from PPMS at 5 and 300 K, respectively. At 5 K, the ferrite nanocrystals show ferrimagnetic behavior with a coercivity of about 300 Oe and the corresponding magnetizations at 30 kOe are 47.4 emu/g for Zn ferrite, 55.7 emu/g for Mn-Zn ferrite, and 62.5 emu/g for Mn ferrite, separately. At 300 K, the nanocrystals become superparamagnetic because of size effects and thermal fluctuations. The inset of Figure 3b reveals the coercivities of all nanocrystals less than 10 Oe. Moreover, the magnetizations of the nanocrystals at 30 kOe are reduced to 30.4 emu/g for Zn ferrite, 37.5 emu/g for Mn-Zn ferrite, and 47.6 emu/g for Mn ferrite, owing to the thermal effects. From the outcomes, it is obvious that the increase of the Mn concentration leads to the increase of the magnetization value. The change in magnetization due to the compositional change may be explained simply by the different moments of the ions, 5 *μ*_B_ of Mn^2+^ ions which are higher than 4 *μ*_B_ of Fe^2+^ ions, in turn 0 *μ*_B_ of Zn^2+^ ions. Other factors such as the inversion parameter in the spinel structure may be considered for comprehensive elaboration of the mechanism. It is useful to remark that the inversion parameter is generally measured by extended X-ray absorption fine structure (EXAFS) analysis or Mössbauer spectroscopy [26,27].

**Figure 4:**
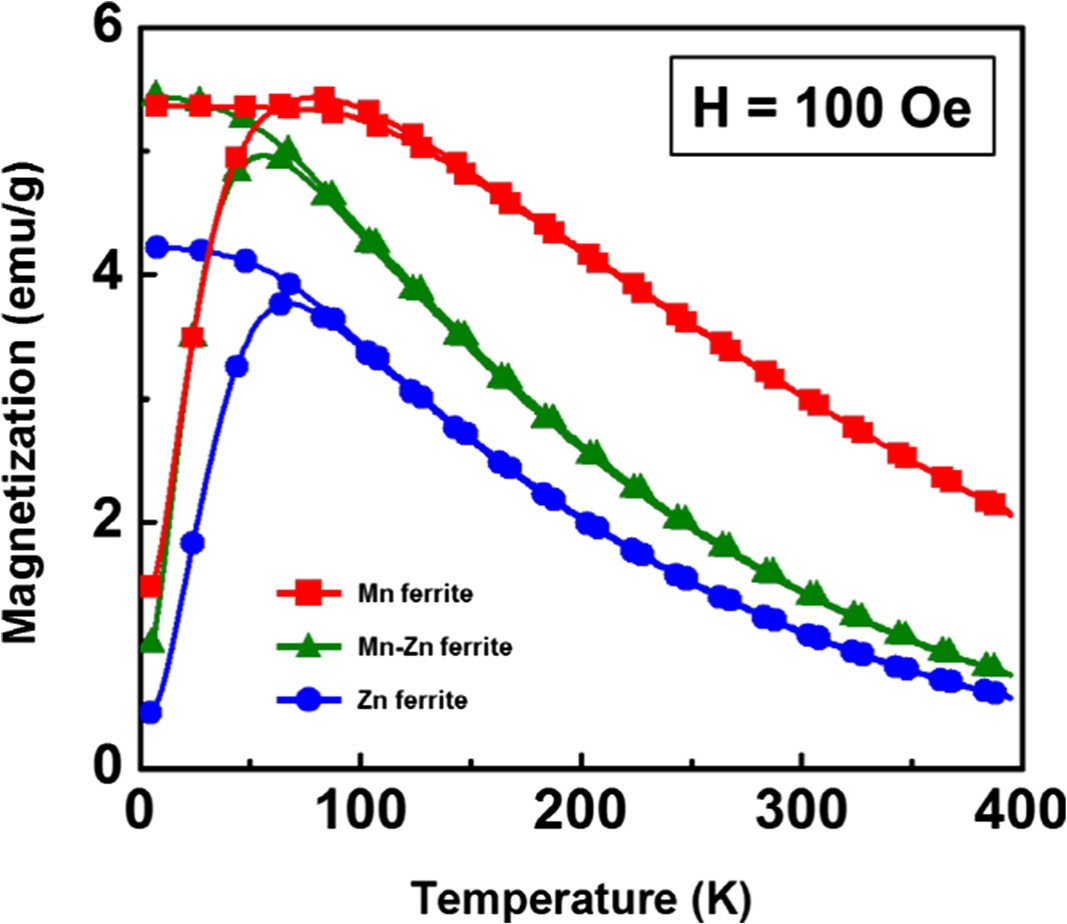
ZFC-FC curves under the magnetic field of 100 Oe for the ferrite nanocrystals.

Furthermore, the temperature dependence of magnetization was recorded in Figure 4 from 5 to 400 K under the applied magnetic field of 100 Oe by the zero-field-cooling (ZFC) and field-cooling (FC) modes. The M-T curves evidently manifest the superparamagnetic behavior of the ferrite nanocrystals. Overall, the magnetization of the nanocrystals in the FC mode decreases gradually as the temperature increases. In the case of the ZFC mode, the magnetic moment of the nanocrystals is frozen to almost zero at the low temperature. With the increasing temperature, the magnetization increases until the blocking temperature (*T*_B_) then decreases like the FC mode. The measured *T*_B_ of the ferrite nanocrystals are 80 K for Mn ferrite, 56 K for Mn-Zn ferrite, and 66 K for Zn ferrite, respectively.

## Conclusions

We have synthesized the ferrite nanocrystals which exhibit high crystallinity and narrow size distributions via the non-aqueous nanoemulsion method and compared three types of samples from Zn ferrite, Mn ferrite, to Mn-Zn ferrites. The structural and chemical measurements performed by XRD and XRF indicated that the ferrite nanocrystals were successfully produced. All samples behave ferrimagnetically at 5 K and superparamagnetically at 300 K, individually. As the concentration of Mn increases, the magnetization value of the ferrites increases. Furthermore, the M-T curves obtained by the ZFC-FC modes clearly substantiate the superparamagnetism of the ferrite nanocrystals.

## Competing interests

The authors declare that they have no competing interests.

## Authors’ contributions

HY and JHM synthesized ferrite nanocrystals and measured microstructure. HY and JSL measured and analyzed the magnetic properties of nanocrystals. This research work was carried out under the instruction of JHW and YKK. All authors contributed to discussing the results and writing manuscript. All authors read and approved the final manuscript.
